# Daily microhabitat shifting of solitarious-phase Desert locust adults: implications for meaningful population monitoring

**DOI:** 10.1186/s40064-016-1741-4

**Published:** 2016-02-01

**Authors:** Koutaro Ould Maeno, Sidi Ould Ely, Satoshi Nakamura, Khemais Abdellaoui, Sory Cissé, Mohamed El Hacen Jaavar, Sid’Ahmed Ould Mohamed, Mohamed Atheimine, Mohamed Abdallahi Ould Babah

**Affiliations:** The Mauritanian Desert Locust Centre, Centre National de Lutte Antiacridienne (CNLA), BP 665, Nouakchott, Mauritania; Hakubi Center for Advanced Research and Laboratory of Insect Ecology, Graduate School of Agriculture Kyoto University, Kyoto, 606-8502 Japan; Japan International Research Center for Agricultural Sciences (JIRCAS), Ohwash 1-1, Tsukuba, Ibaraki 305-8686 Japan; Département des Sciences Biologiques et de la Protection des Végétaux, Institut Supérieur Agronomique de Chott-Mariem, BP 47, 4042 Chott-Mariem, Sousse, Tunisia; Centre National de Lutte contre le Criquet pèlerin, BP E-4281, Quartier du fleuve, Rue 313, Porte 261, Bamako, Mali

**Keywords:** Defensive behaviour, Density-dependent phase polyphenism, Monitoring, *Schistocerca gregaria*, Solitarious locusts, Thermoregulation

## Abstract

The Desert locust *Schistocerca gregaria* is a major world pest that causes substantial agricultural and economic damage. Effective pest control relies on effective monitoring, which requires knowledge of locust microhabitat selection. Yet little is known about microhabitat selection of solitarious adult locusts in the field. We conducted field surveys to investigate fine-scale diel temporal and spatial distributions of solitarious adults in the Sahara Desert in Mauritania, a major breeding and recession area. We found that solitarious adults moved among different, specific microhabitats throughout the 24-h period in a cyclical manner. At night, they roosted in trees, moved to the ground to feed shortly after dawn, sheltered in low vegetation during the hot midday, and returned to the ground in the late afternoon. Hence, they switched microhabitats and plant species throughout each day. These cyclical daily movements among diverse microhabitats and specific plant species were correlated with time of day, light intensity, temperature, humidity, and specific plant species, and may relate to anti-predator defence, thermoregulation, and feeding. The present study suggests that locust monitoring should be adjusted, based on time of day, locust age, phase state and relative abundance of specific plant species. For example, we recommend surveying ground after morning and trees at night, for solitarious adults, when at low density.

## Background

The Desert locust *Schistocerca gregaria* (Forskål, 1775) is one of the most destructive pests in the world (Sword et al. 2010). The species exhibits phenotypic plasticity, known as density-dependent phase polyphenism, whereby individuals can exist anywhere between two extreme forms (solitarious vs. gregarious), which differ in morphology, physiology, behavior, ecology, and propensity to cause agricultural damage (Simpson and Sword 2009; Wilson and Cotter 2009). The solitarious form occurs at low population density and is sedentary and relatively harmless. When solitarious locusts increase in population density, they begin to progressively gregarize and change their behavioral, morphological and physiological characteristics, in a process (Uvarov 1966; Applebaum and Heifetz 1999; Pener and Simpson 2009; Simpson and Sword 2009). Gregarized locusts tend to aggregate, form swarms consisting of several billion individuals, and devastate crops (Uvarov 1977; Sword et al. 2010). Full gregarization usually needs at least two generations for destructive plagues to appear, such as the historical 2003–2005 outbreak in West Africa (Brader et al. 2006).

The primary pest control method used against the Desert locust is detection and prevention. In this method, wide areas of Africa are monitored to find locust populations that may be gregarizing. The goal is to find and treat such populations with insecticides while they are still in limited number and density, preferably in the nymphal stages, before they reach adulthood and can migrate as swarms to agricultural areas (Brader et al. 2006; Cressman 1996; Lecoq 2001; Magor et al. 2008; Sword et al. 2010; Babah 2011).

Outbreaks of Desert locust begin when solitarious locusts encounter other individuals at limited favorable habitats; therefore, monitoring for distribution and abundance of solitarious populations to detect local gregarization is essential to develop an early warning system (van der Werf et al. 2005; Sword et al. 2010). However, *S. gregaria* can breed across a vast geographical area of 31 million km^2^ (Lecoq 2004). It is nearly impossible to inspect this entire area each year. But, scientists have identified specific environmental conditions that predict possible locust outbreaks, on a coarse scale, such as rainfall and temperature patterns, and resultant vegetative growth (Sword et al. 2010). Also, specific “locust-breeding areas” have been identified in some countries such as Mauritania (Babah 2011; Piou et al. 2013). As a result, technicians can use weather data and remote sensing to identify potential hot-spots, in addition to a massive information collection from all resources (Nomads and militaries etc.), and then selectively inspect such areas. Such methods examine locust distribution and ecological conditions on a course geographic scale. However, within a specific population or locality, locusts may distribute themselves in a finer scale, such as specific microhabitats or specific plant species, within the larger habitat. Although, knowing the fine-scale distribution of locusts might aid pest control (Lecoq 2005), information is very limited. This is especially true for solitarious *S. gregaria* adults, because their low population density, remote and inaccessible habitat, extreme environmental conditions, and high flight mobility makes their study difficult (Kennedy 1939; Ellis and Ashall 1957; Roffey and Popov 1968; Uvarov 1977; Culmsee 2002).

Mauritania is a major breeding and recession area for the Desert locust (Babah 1997, 2010; Babah and Sword 2004). The Mauritanian Desert Locust Centre (CNLA), a member of the Locust Commission in Charge of Desert Locust Coordination in Western Region (CLCPRO) in Africa, developed a real-time monitoring system to follow locust field population dynamics based on highly mobile ground survey teams equipped with long-range radios (Babah 2011). Support systems for field surveys allow researchers to remain at field sites for long periods. We used this system to access a suitable survey site within the natural Desert locust habitat in Northwestern Mauritania, where locust population density was low and vegetation was abundant and green. The purpose of the present study was to discover the fine-scale distribution of solitarious adult *S. gregaria*, and to understand their diel patterns of microhabitat selection, in order to develop better survey and treatment methods.

## Results

### Phase state of adult locusts

**Fig. 1:**
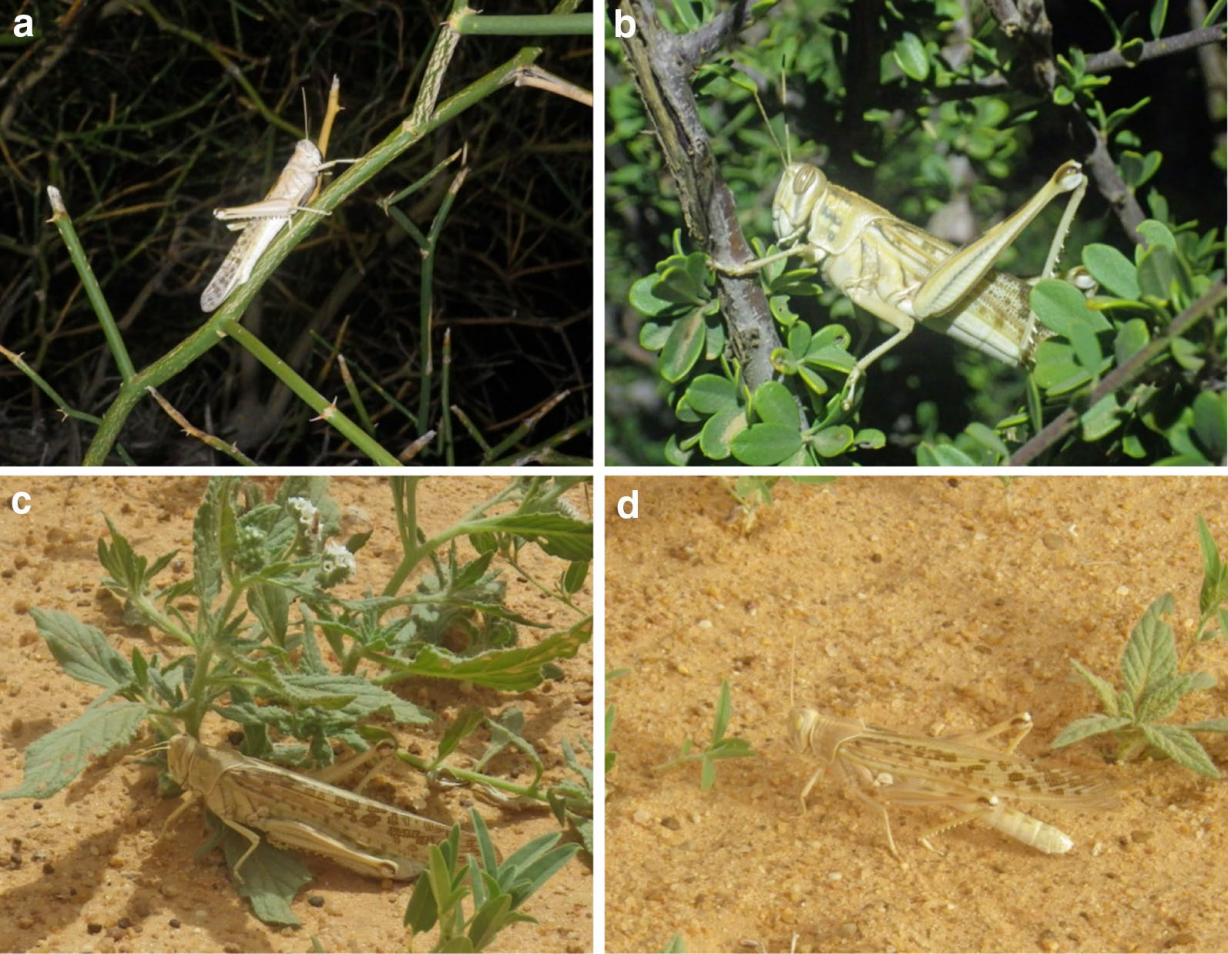
Solitarious adult *Schistocerca gregaria* switched microhabitats throughout the day in a population in Northwestern Mauritania. During the night, they roosted in **a** *Capparis decidua* or **b** *Maerua crassifolia*. **c** During the hot mid-day, they often shaded under plants. **d** During early and mid-morning and late afternoon, they mainly occupied open bare ground. Note that solitarious adults were well camouflaged against the desert soil

In this study, population densities in individual 100 m^2^ belt transects ranged from a low of 0 individuals/m^2^ to a high of 0.06 individuals/m^2^ (6 individuals/ha), with an overall mean density of 0.0023 (SE = ±0.0003) individuals/m^2^ (*n* = 225 transects). Hence, the locusts in our study were at a relatively low density, and adults were generally widely spaced, with virtually no aggregation (Fig. 1).

Ninety-nine percent of the *S. gregaria* found were adults (*n* = 116). Their bodies were hard and light brown in color, their hind wings were yellowish (*n* = 58), and 2 % were in copulation. Six out of 26 females examined had egg pod foam on their ovipositor valves, indicating that they had already laid eggs. Hence, the average age of the population might be at least 2 weeks after the adult emergence.

The percentage of adults with six nymphal stadia (7 eye stripes) was 69.2 (*n* = 26) and 28.1 (*n* = 32) for females and males, respectively. Collected locusts had relatively high F/C ratios, indicative of solitarious phase. The mean F/C ratio of adults with five nymphal stadia was 3.97 (SE = ±0.04, *n* = 8) and 3.93 (SE = ±0.02, *n* = 23) for females and males, respectively. Some adults had undergone six nymphal stadia and their F/C ratio was 4.08 (SE = ± 0.03, *n* = 18) and 4.07 (SE = ±0.04, *n* = 9) for females and males, respectively. These morphological, behavioral and physiological characteristics indicate that our locust population consisted of solitarious phase sexually mature adults.

### Daily cyclical microhabitat selection

**Fig. 2:**
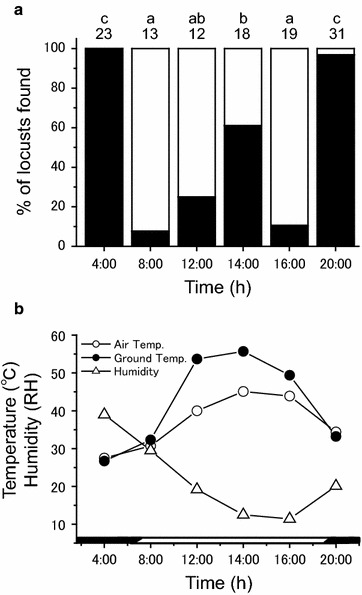
**a** Percentage of solitarious adults of *Schistocerca gregaria* found in different microhabitats either on the ground (*white*) or on plants (*black*). **b** Diel environmental factors including air temperature (*open circle*), ground surface temperature in sunlight (*filled circle*) and relative humidity (*open triangle*) on a typical sunny day (30 September, 2013) at Targa, Northwestern Mauritania. *Dark bar* along *horizontal axis* represents night time. Different letters on the bars indicate significant differences at *P* < 0.003 between values (post hoc Fisher’s exact test after Bonferroni correction). Numbers above bars indicate sample sizes

**Fig. 3:**
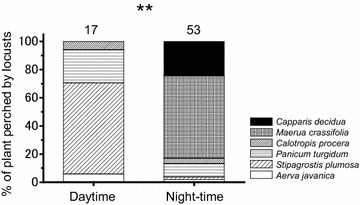
Percentage of six different plant species (tree: *Capparis decidua* and *Maerua crassifolia*; shrub: *Calotropis procera*; bush: *Panicum turgidum* and *Aerva javanica*; grass: *Stipagrostis plumose*) occupied (perched on) by solitarious adult *Schistocerca gregaria* at daytime or night-time. Numbers above bars indicate sample sizes. Asterisk in the graph indicates significant difference between the two groups (χ^2^-test; **P < 0.01)

Figure 2 shows the vertical distribution (ground vs. plants) of locusts at different times of the day, and under different environmental conditions. The locusts showed strong diel movements among microhabitats. During night, locusts mainly roosted on two tree species (Figs. 1a, b, 3). At dawn, the locusts solar-basked, by orienting their bodies perpendicular to the sun’s rays. Shortly after, as sunlight intensity and air temperature rapidly increased (Fig. 2b), they moved to relatively open, sunny ground (Fig. 2a), where they fed on low-growing annuals. At midday, under intense solar radiation and as ground temperatures surpassed 50 °C, locusts moved into shade, with 40 % roosting off the ground, in shady plants (Figs. 1c, 2). In the late afternoon, as air and ground temperatures fell, the locusts returned to the sunny ground to feed (Fig. 1d). Finally, in the late afternoon, before sunset, as light intensity declined, the locusts flew or walked to sometimes distant trees. They ascended the trees, and remained there until dawn (Fig. 2a). Hence, solitarious *S. gregaria* adults exhibited daily cyclical microhabitat shifts between plants, ground, sunlight, and shade (Figs. 2a, b).

**Fig. 4:**
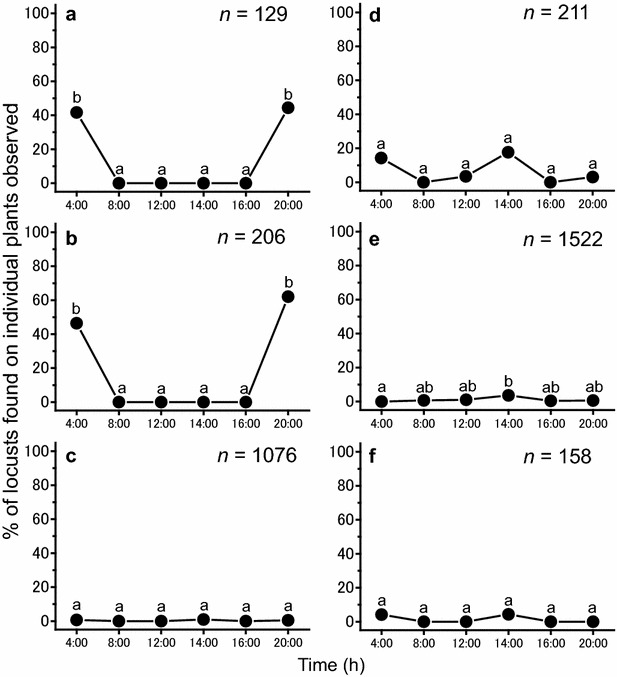
Diel changes of plant utilization patterns by solitarious adults of *Schistocerca gregaria* on each of six plant species including. *Graphs* show percentages of locusts found on individual plants observed: **a** *Capparis decidua*, tree; **b** *Maerua crassifolia*, tree; **c** *Calotropis procera*, shrub; **d** *Panicum turgidum*, bush; **e** *Stipagrostis plumose*, grass and **f** *Aerva javanica*, bush. *Different letters* on the *bars* indicate significant differences at *P* < 0.003 between values (post hoc Fisher’s exact test after Bonferroni correction). Numbers in the graphs indicate sample sizes

In addition to moving between plants and ground, sun and shade, the locusts also cycled among specific plant species throughout the day. Table 1 shows the characteristics of six large-size plant species. The large trees, *C. decidua* and *M. crassifolia*, were relatively scarce (Table 1), but their height allowed them to be seen from afar. The other plants were significantly smaller in size than the trees, but tended to be more abundant (e.g., *C. procera* and *S. plumosa*) (Tukey–Kramer test, *P* < 0.05). During nights, the locusts roosted in the large, but relatively scarce, trees, *C. decidua* and *M. crassifolia* (Figs. 1a, b, 3). During mornings and afternoons, they used low-growing annuals such as *T. alatus* and *H. ramosissimum* as food plants, and during the hot mid-day, they mainly roosted on grasses such as *S. plumosa* and the bush *P. turgidum* (Figs. 3, 4) (χ^2^-test, χ^2^ = 48.189, *P* < 0.01). Interestingly, locusts tended to avoid trees during the day, but roosted in them during the night. Hence, frequency of plant utilization patterns of solitarious adults varied throughout the day, depending on the plant species (Fig. 4).

**Table 1:** Mean (±SE) number of plants per 100 m^2^ belt transect (*n* = 402 transects total) and plant sizes (m^3^) of six dominant plant species in the survey site (35 individuals measured per plant species)

Plant species	Types of plant		No. of plants (100 m^2^)	*n*	Plant size (m^3^)	*n*
*Capparis decidua*	Perennials	Tree	0.16 ± 0.02 a	402	53.28 ± 9.78 c	35
*Maerua crassifolia*	Perennials	Tree	0.27 ± 0.02 bc	402	19.54 ± 3.28 b	35
*Calotropis procera*	Perennials	Bush	1.34 ± 0.14 d	402	7.31 ± 2.78 ab	35
*Panicum turgidum*	Perennials	Bush	0.26 ± 0.03 bc	402	3.68 ± 0.47 ab	35
*Stipagrostis plumosa*	Annuals	Bush	1.89 ± 0.13 e	402	0.97 ± 0.11 a	35
*Aerva javanica*	Annuals	Bush	0.20 ± 0.12 b	402	0.64 ± 0.11 a	35

Different letters after values indicate significant differences between values within a column (Tukey–Kramer test, *P* < 0.05)

Locusts also varied their escape behavior throughout the 24-h period. During the night, tree-roosting locusts were slow to escape, and could be approached and caught by hand. However, escape behavior changed during the day. Between 08:00 and 16:00, ground inhabiting locusts quickly flew away when human observers approached to within 5 m. Such locusts typically flew ~30 m. The majority of locusts (99.0 %, *n* = 132) flushed in this manner landed on the ground, but their subsequent behavior varied with ground temperature and time of day. At 08:00 (100 %, *n* = 51) and 16:00 (100 %, *n* = 30), all flushed locusts remained stationary for more than 3 min after landing on the ground. In contrast, at 14:00 all (100 %, *n* = 51) flushed locusts quickly moved from the ground by hopping or walking to nearby plants or shade, and then became stationary after reaching this microhabitat (Fig. 1c). At 18:00, three flushed males remained unmoving on the ground for more than 20 min. This suggests that locusts vary their defence behavior and microhabitat preference depending on ground temperature. During mid-day, when soil temperatures are high, they immediately move away from the ground. In early morning and late afternoon, when ground temperatures are cooler, they remain motionless. The solitarious adult locusts in our study were well camouflaged when on the ground, but less so when on vegetation (Fig. 1a–d).

It was difficult to count and to collect locusts during the day. After locusts became warm, they occupied ground, where they were well camouflaged (Fig. 1d). Such locusts would typically take flight 1–10 m from an approaching human, and typically fly ~30 m. As such, it was often impossible to tell if the flying insect was a locust or a different species of insect for non-experts, and if the locust had been inside or outside the belt transect. In contrast, one could easily approach and count, or collect locust that were roosting at night.

## Discussion

In our study, solitarious-phase adult *S. gregaria* from Northwestern Mauritania actively moved among diverse microhabitats throughout the day. These cyclical microhabitat shifts were correlated with daily photoperiod and temperature cycles, and specific plant species. This knowledge not only aids our understanding of locust biology and ecology, but is important for effective locust control.

Many grasshopper species are known to cycle among divergent microhabitats during each 24-h period (Uvarov 1977). This is especially true for adults of desert inhabiting species (Waloff 1963; Uvarov 1977; Whitman 1987, 1988; Symmons and Cressman 2001). Although desert grasshoppers exhibit diverse behaviors associated with phylogeny, phase state, and current physiological and environmental conditions (Roffey and Popov 1968; Uvarov 1977; Stauffer and Whitman 1997), they generally shelter off the ground in plants during the cool night. At sunrise, they solar-bask on the plants, quickly warm, and then descend to the ground to feed. During midday, when ground temperatures become intolerably high, they move back into the relatively cooler vegetation, where they seek shade and shelter from the sun. The locusts are very adept at locating and using tiny patches of shade, during the hot midday. When the air and ground temperatures fall in the late afternoon, the insects once again move to the ground, before ascending plants for the night (Uvarov 1977; Chappell and Whitman 1990). We observed a similar daily cyclical activity pattern for solitarious adult *S. gregaria* and noted that their microhabitat selection was closely associated with specific plant species.

The present study confirmed that in Northwestern Mauritania, solitarious adults differ dramatically from solitarious nymphs in diel patterns of microhabitat shifts. Adults moved readily among microhabitats and different plant species throughout the day, and generally roosted in trees at night. In contrast, solitarious nymphs tended to use a single species of plant, the spiny perennial herb *Fagonia arabica* (Zygophyllaceae), as a food and nocturnal shelter at a certain site (Maeno et al. 2012). In addition, solitarious adults spent large portions of the daytime on the ground, whereas solitarious nymphs tended to remain on their host plant throughout the entire 24-h period (Maeno unpublished observation). This may be because it is less dangerous for the winged adults to change microhabitats, as they can escape heat and predators by immediately flying away. In addition, solitarious nymphs tend to be camouflaged against plants (Stower 1959), whereas solitarious adults are better camouflaged against the ground.

Another important point is that gregarious phase *S. gregaria* behaves quite differently from solitarious phase *S. gregaria* (Simpson and Sword 2009; Ould Ely et al. 2011). Gregarious nymphs often occupy the ground for long periods, forming large and dense aggregations that move in mass (Ellis and Ashall 1957; Culmsee 2002; Maeno et al. 2013). Solitarious nymphs behave in an opposite manner: they generally do not aggregate, nor move to the ground, but tend to remain on a specific species of plant throughout the day. Likewise, solitarious adults do not aggregate when host plants are abundant (Cisse et al. 2013), whereas gregarious adults aggregate and move in dense swarms (Uvarov 1977).

### What is the function of cyclical microhabitat shifting?

Daily microhabitat shifting in *S. gregaria* probably functions in anti-predator defence, thermal regulation and feeding. Grasshoppers, including *S. gregaria* are attacked by diverse predators, including insects, spiders, reptiles, birds and mammals (Greathead 1963; Joern and Gaines 1990; Whitman and Vincent 2008; Ould Ely et al. 2011). Predator type (e.g., mammals vs. birds; invertebrate vs. vertebrate predator) and predator-specific mortality changes throughout the 24-h period (e.g., nocturnal vs. diurnal predators), among different microhabitats (ground vs. grasses vs. trees), and as the grasshoppers grow (nymphs are often eaten by invertebrates, and adults by vertebrate predators) (Greathead 1963; Uvarov 1977; Whitman 1988; Whitman and Vincent 2008). Because the day is so hot, many desert predators, such as the Mauritania’s insectivorous jerboas (*Jaculus jaculus*) (Dipodidae), are nocturnal, hunting on the ground and in low vegetation at night (Jaeger 1961; Crawford 1981; Scott et al. 2005). As such, the ground is a dangerous place for desert grasshoppers at night, and we observed very few locusts on the ground at this period (Fig. 2). This may be why adult locusts fly to and roost in the tallest nearby trees before night, although such trees are significantly less abundant than other types of plants (Table 1). Nocturnal tree-roosting cannot be for food purposes, because locusts seldom feed on either of these tree species (Fig. 3). In fact, the adults often nocturnal-roost on bare, dead branches (Fig. 1a). Roosting high in trees allows locusts to escape nocturnal ground predators. Although some nocturnal predators do hunt in trees, the size, complex branching, and three-dimensional architecture of trees makes it hard for nocturnal arboreal predators to find locusts, compared to on the two-dimensional ground. In addition, tree-roosting locusts employ a backup strategy when found by a nocturnal predator; they simply drop to the ground. They can also fly when night temperature is high enough (Uvarov 1977). It is difficult for nocturnal arboreal predators to find locust prey, once they have dropped to the ground. Although locusts shelter in trees at night, at midday, they select a different microhabitat for shelter. At midday, locusts shelter in low grasses, shrubs and bushes. Selecting low vegetation during midday may allow locusts to avoid birds (a primary diurnal predator), which commonly shade in trees during midday. Thus, daily microhabitat shifts may aid anti-predator defence.

Cyclical microhabitat shifting may also aid thermoregulation. Desert locusts thermoregulate by basking in sunlight, when cold (Waloff 1963). Roosting high in trees may facilitate such radiative heating (Whitman 1987, 1988). Tree tops are the first microhabitat to receive sunlight at dawn. In contrast, a locust on the ground would not only receive direct sunlight later, but would heat more slowly because of thermal inertia of the cool dawn soil. However, as the sun rises, the desert ground heats rapidly, and by midday often becomes lethally hot. According to various authors, Desert locusts enter heat torpor at about 49 °C (see Table 6.1 in Chappell and Whitman 1990). As such, on warm days, Desert locusts must leave the ground microhabitat well before midday, as observed for some other desert grasshoppers, such as *Taeniopoda eques* (Whitman, 1987).

Daily microhabitat cycling in locusts may also relate to feeding, mating, oviposition, and local dispersal. Locusts must feed, but they generally shun the large roosting-plants. Instead, locusts prefer to eat low-growing annuals. Hence, to feed, locust must switch microhabitats. When feeding, adult solitarious locusts typically wander along the ground, sampling numerous plant species. Consuming a diversity of plant species, may allow them to balance nutrients and dilute specific plant toxins (Chambers et al. 2001). In the desert, the best conditions for ground-feeding are usually in the morning and late afternoon. At night, cooler temperatures lower feeding efficiency or, in extreme conditions, prevent feeding due to cold-torpor (Chappell and Whitman 1990), and, as previously noted, nocturnal ground feeding may be disadvantageous because of nocturnal ground predators. Likewise, ground feeding is usually impossible at midday due to high stressful ground temperatures. Thus, daily microhabitat shifts in locusts may relate to favorable periods and locations for feeding.

Ground occupancy during morning and late afternoon probably also relates to dispersal, mating, and oviposition. For example, sexually mature males usually search on the ground for ground-feeding females, and females must be on the ground for oviposition into soil (Stauffer and Whitman 1997).

Local gregarization of solitarious adults occur at limited favorable areas when vegetation start to dry up at the end of a breeding season (Symmons and Cressman 2001). The present study observed that solitarious adults selectively roosted on trees at night. This strong microhabitat preference might lead to local crowding on specific plant species before starting dry season. In addition, solitarious adults tended to move to Eastern side of roosting tree where the first microhabitat to receive sunlight at dawn to bask. This thermoregulatory behavior will force solitarious adults to concentrate at a very limited site even population density is very low, as observed by Roffey and Popov (1968). Thus, a combination of microhabitat selection and thermoregulatory behavior of solitarious adults might play an important to role in the process of gregarization via local crowding. If so, the spatial distribution of overnight roosting trees could be used assess the relative risk of certain habitats promoting the gregarization of solitarious adult population at given densities. It has been reported that solitarious adults fly during an early part of the night (Roffey and Popov 1968). Understanding of this night flying of solitarious adults also important to develop monitoring system.

### Locust control and population monitoring

Locust control depends on population monitoring—first finding, then censusing and then treating those specific populations that are increasing in density or becoming gregarious (Sword et al. 2010). This is a difficult task in affected countries, an area of over 31 million km^2^ (Lecoq 2004). However, searching for locust populations is not random, because plant community influences both large-scale geographic distribution and local distribution of locusts (McCulloch and Hunter 1983; Woldewahid et al. 2004; van der Werf et al. 2005). Specifically, *S. gregaria* distribution and probability of gregarization are closely related to plant community (Babah 2010; van der Werf et al. 2005; Cisse et al. 2013, 2015). Remote sensing to detect green vegetation, which is essential for breeding, is one tool used to monitor the vast remote areas of North Africa (McCulloch and Hunter 1983; Cressman 1996, 2013; Piou et al. 2013). Once probable locust areas are identified, workers must still locate the specific locust-sites, and this can be aided by knowledge of their preferred microhabitats.

Reliable censusing of locust field populations is essential for locust pest managers and researchers. But to accurately census locusts, one must know where they are, i.e., their fine-scale distribution, including the specific plants and microhabitats inhabited by locusts at different times of the day. Our research shows that solitarious adult locust switch microhabitats throughout the 24-h period. Locust experts must accommodate such diel shifts into their sampling strategies. For example, it is common to survey locust populations by line transects that count the number of locusts on the ground during daytime (Cressman 2001; Babah and Sword 2004). The present results clearly suggest that this method should be avoided at early morning and at dusk, because locusts tend to hide inside plants at this time. Also, as previously mentioned, it is difficult to accurately survey or collect solitarious adults during the day, because they readily fly away before they can be approached, and because their extremely low density requires extensive search efforts to find few locusts. Therefore, we recommend that adult solitarious locusts be surveyed and collected at night from large trees or bushes. At this time of day and in this microhabitat, not only are the locust aggregated, but they are relatively immobile, and thus can be closely approached. Also, large trees are easily located in the habitat. This technique is efficient, in that it can reduce effort and time for monitoring. In particular, the density (and spatial distribution) of the trees present in a given habitat must also be accounted for. More trees in a given area will presumably result in fewer solitarious adult locusts per tree, and vice versa. Thus, if monitoring teams are to sample trees at night, they must have protocols developed to sample both locusts at night as well as the distribution of trees in the same habitat. Plus, these two measures then need to be combined into a useful metric of locust density.

## Conclusion

Our current paper has important implications for locust monitoring and control. We focused on the fine-grain distribution of solitarious adult locusts, within a small geographic area. We show that within a single local population, locust distribution is influenced by plant species, locust age, time of day, and various environmental and meteorological factors. We also demonstrate that locusts move among different specific plants and microhabitats in a cyclical manner within a day. These findings have important practical implications for those who survey and treat locust populations in the field. They suggest that survey and treatment strategies should be adjusted based on time of day, locust age, phase state, and relative abundance of specific plant species (e.g., trees vs. grasses) and temperatures. Specifically, we recommend that locust workers survey trees and large bushes at night. The results also affirm that both macro- and micro-monitoring techniques would contribute to develop preventative controls. Lastly, we remind readers that locust behavior is extremely variable. They exhibit different behaviors, including microhabitat occupancy, depending on ontogenetic stage, phase state, physiological state, population density, vegetation characteristics, and weather. Locust workers need to understand this behavioral plasticity and incorporate it into their research and pest control strategies and methods.

## Methods

### Study area

Mauritania, in West Africa, is an important area in which gregarization occurs within the recession zone of the Desert locust (Babah 1997, 2010). The study site (N18°32′, W15°04′) is located in Targa, Northwestern Mauritania. This area has no permanent human population and access is difficult. The area consists primarily of sparse desert (mixed grass and desert shrubs with occasional trees), and has six dominant (= large size) plant species: the bush-like bunch-grass, *Panicum turgidum* (Poaceae), the bushes, *Calotropis procera* (Asclepiadaceae), *Stipagrostis plumosa* (Poaceae), and *Aerva javanica* (Amaranthaceae), and the trees, *Capparis decidua* (Capparaceae), and *Maerua crassifolia* (Capparaceae). These plants occur in discontinuous patches. Numerous short forbs such as *Tribulus alatus* (Zygophyllaceae), *Boeravia repens* (Nyctaginaceae), *Heliotropium ramosissimum* (Boraginaceae), *Farsetia stylosa* (Brassicaceae), *Gisekia phanacoïdes* (Molluginaceae) are also present, and many of these grow as thick clumps, with numerous, dense stems arising from a root mass (Duranton et al. 2012). In general, the six dominant plants covered about 20 % of the soil surface, but plant cover ranges from bare ground to ~90 % cover (Monod 1954). The six dominant plant species generally are not eaten by *S. gregaria*. Instead, locusts prefer to eat low-growing annuals, such as *T. alatus* and *H. ramosissimum*. We conducted field surveys during September 2013. Sunrise and sunset at local time was about 0650 and 1900 h, respectively. Typical daily temperatures and humidities are shown in Fig. 2.

### Sampling regime

The survey site (1 km × 1 km) was flat and was randomly chosen from the larger area. To determine population densities (number of locusts/m^2^) we conducted 225 belt transects (each 2 × 50 m) between 08:00–18:30. Transects were separated by at least 10 m. To monitor microhabitat shifting in locusts, we recorded microhabitat distribution (open ground, sheltering under plant, roosting on plant or within dense vegetation, etc.) by solitarious *S. gregaria* adults, at 04:00, 08:00, 12:00, 14:00, 16:00 and 20:00 during similar 50 m transects, as above. Each sampling period (e.g., 12:00, 16:00, 20:00, etc.) required about 2.5 h to complete. Transects were conducted on 18, 19, 29 and 30 September 2013 and data were pooled. For each transect, we recorded shaded air temperature at 50 cm above the ground, surface temperature of sunlit soil surface, and relative humidity in shade at 50 cm. We also recorded number of locusts found, their vertical location (ground vs. in plant), and the plant species occupied. At least 10 belt transects (2 × 50 m) surveys were conducted at each sample period within the 1 km^2^ survey site. All medium and large plants (shrubs, trees, and clumps of bunch-grass with diameters >10 cm) within each transect were closely inspected to see if locusts sheltered in or under them. A total of 3302 medium and large plants were observed within 402 transects. Because of the scarcity of the trees *C. decidua* and *M. crassifolia*, additional transects that included these plants were surveyed to compensate for the small sample size. We used red lights for night observation.

### Plant size and plant abundance

We used a tape measure to measure the maximum length, width and height of 35 of each of the six dominant plant species, as per Maeno et al. (2012). The volume of each plant was estimated as the product of these measurements (maximum length × width × height = volume in m^3^). Plants less than 10 cm in any one of the dimensions were not measured.

### Morphometric measurements

We collected 58 solitarious adults by sweep nets from larger roosting-plants, at night, and then measured the hind femur length (F) and maximum head width (C), using digital calipers (SC-15S, Mitsutoyo Co., Tokyo, Japan) to determine the classical morphometric ratios of F/C (Dirsh 1951).

*S. gregaria* goes through five or six nymphal stadia before reaching the adult stage (Roonwal 1947). Adults with five versus six nymphal stadia have six versus seven eye stripes, respectively. The number of nymphal stadia influences the F/C ratio at the adult stage (Maeno and Tanaka 2008). Therefore, adults with six or seven eye stripes were analyzed separately.

### Age determination

We estimated the age of locusts collected, by three criteria: body hardness, hind wing color, and evidence of recent oviposition (in females only). The cuticle is soft in 6-day-old solitarious adults, but becomes harder as they age (Duranton and Lecoq 1990). Likewise, the hind wings of 6-day-old solitarious adults are transparent, but become yellow when locusts become sexually mature (Norris 1954; Pener 1991). Female adults produce foam to plug a digging hole during oviposition. The presence of foam adhering to the ovipositor valves indicates that adult females have already deposited eggs at least once, and therefore must be at least 15-day-old.

### Microhabitat selection related to solar intensity and temperature

Locusts use plants and shade as shelter during extremely high midday temperatures (Uvarov 1977). To observe microhabitat selection associated with the thermoregulation behavior of avoiding overheating for solitarious adults, we employed the following method (Maeno et al. 2013): at three different times of the day (08:00, 14:00 and 16:00): a researcher dressed in white walked through the habitat. This caused adults to fly and land on the ground. Such locusts were observed for 3 min, to determine their subsequent behavior (remaining stationary or moving to shelter, either plant or shade). This procedure was repeated twice for each time period.

### Statistical analysis

Data for females and males were pooled. The number of locusts roosting on plants was analyzed to compare aggregation from sunrise to sunset, and from sunset to sunrise, respectively. Tukey–Kramer tests were conducted to analyze significant differences among number of plants and sizes. Percentages of locusts found on plants were subjected to a post hoc Fisher’s exact test after Bonferroni correction by using the software package R, version 3.1.1 (R Development Core Team 2012). We did not statistically compare locusts numbers observed among different times, because we conducted different numbers of belt transects at different times.
